# Structural and biochemical analyses of a novel bacterial dual specificity phosphatase from *Candidatus Chlorohelix allophototropha*

**DOI:** 10.71150/jm.2604025

**Published:** 2026-07-16

**Authors:** Sujin Jung, So Hyeon Park, Joon Sig Choi, Ho-Chul Shin, Seung Jun Kim, Bonsu Ku

**Affiliations:** 1 Orphan Disease Therapeutic Target Research Center, Korea Research Institute of Bioscience and Biotechnology, Daejeon 34141, Republic of Korea; 2 Department of Biochemistry, Chungnam National University, Daejeon 34134, Republic of Korea; 3 Critical Diseases Diagnostics Convergence Research Center, Korea Research Institute of Bioscience and Biotechnology, Daejeon 34141, Republic of Korea

**Keywords:** dual specificity phosphatase, protein tyrosine phosphatase, DUSP, PTP, *Candidatus Chlorohelix allophototropha*, *CCa*DUSP, crystal structure

## Abstract

Dual specificity phosphatases (DUSPs) are a subfamily of protein tyrosine phosphatases that regulate diverse cellular processes through dephosphorylation of phosphorylated substrates. DUSPs are commonly found in eukaryotes, bacteria, archaea, and viruses. However, structural and biochemical characterization of bacterial DUSP remains limited, as only one bacterial DUSP has been identified thus far. In this study, we investigated a novel putative bacterial DUSP from *Candidatus Chlorohelix allophototropha*, referred to as *CCa*DUSP. The crystal structure of *CCa*DUSP showed the presence of a well-conserved catalytic motif with a characteristic phosphate-binding loop. Biochemical analyses further confirmed that *CCa*DUSP exhibits phosphatase activity and contains dual general acid/base residues, both of which contribute to its enzymatic activity. These findings not only represent the first characterization of a novel bacterial DUSP with dual general acid/base residues but also provide a foundation for understanding the diversity of DUSP proteins in bacteria.

## Introduction

Protein phosphorylation is a key post-translational modification involved in the regulation of various cellular processes. Cellular phosphorylation levels are tightly controlled by the coordinated action of kinases and protein phosphatases (Tonks, 2006). The protein tyrosine phosphatase (PTP) family consists of diverse enzymes that catalyze the removal of phosphate groups from phosphorylated substrates (Alonso et al., 2004). Dual specificity phosphatases (DUSPs) constitute a subfamily of PTPs that are capable of dephosphorylating both phosphoserine/phosphothreonine and phosphotyrosine residues (Patterson et al., 2009). DUSP proteins possess a conserved catalytic domain containing the signature phosphate-binding loop (P-loop) motif, H−C−x−x−G−x−x−R−S/T (Jeong et al., 2014). DUSPs constitute the sole PTP subfamily commonly found in eukaryotes, bacteria, archaea, and viruses (Ku, 2025). To date, only one bacterial DUSP, TpbA from *Pseudomonas aeruginosa*, has been structurally and biochemically characterized (Koveal et al., 2013; Xu et al., 2015). Recently, we reported the presence of an additional putative bacterial DUSP protein in *Candidatus Chlorohelix allophototropha* (Ku, 2025), an anoxygenic phototroph in the Chloroflexota phylum (Tsuji et al., 2024). Sequence alignment of this protein, referred to as *CCa*DUSP in this study, suggests that it shares similarities with several representative DUSP family members, including *P. aeruginosa* TpbA (a bacterial DUSP), *Thermococcus kodakaraensis* Tk-PTP (an archaeal DUSP), and human DUSP23a (Fig. 1A). In the present study, we investigated the crystal structure and enzymatic characteristics of *CCa*DUSP. We demonstrated that it harbors a well-conserved catalytic motif and dual general acid/base residues, providing a basis for understanding the diversity of DUSP family proteins in bacteria.

## Materials and Methods

### Preparation, crystallization, and structural determination

The coding region corresponding to residues 1–160 of *CCa*DUSP (RefSeq: WP_341469495.1) was subcloned into a modified pET28a vector (Novagen) fused in frame with an His_10_−maltose-binding protein tag at the N-terminal region. The *CCa*DUSP recombinant protein was expressed in *Escherichia coli* BL21(DE3) RIL cells (Novagen) cultured in Luria–Bertani medium at 25°C for 12 h following induction with 0.1 mM isopropyl β-D-thiogalactopyranoside. The protein was purified using Ni-NTA affinity chromatography (QIAGEN), followed by size-exclusion chromatography on a HiLoad 26/600 Superdex 75 prep grade column (Cytiva). The His_10_−maltose-binding protein tag was removed during purification after cleavage with tobacco etch virus protease. Protein samples were equilibrated with running buffer containing 50 mM Tris–HCl (pH 7.5), 200 mM NaCl, and 2 mM dithiothreitol. Crystallization trials were set up using the sitting-drop vapor diffusion method at 18°C by mixing 1 μl of protein solution (20 mg/ml) with 1 μl of precipitant solution, which is composed of 0.1 M Tris–HCl (pH 8.5), 1.5 M ammonium sulfate, and 12% glycerol. Diffraction data were collected at Beamline 5C of the Pohang Accelerator Laboratory (Korea) and processed using *HKL*2000 (Otwinowski and Minor, 1997). Molecular replacement was carried out using Phaser (McCoy et al., 2007) with an AlphaFold3-predicted model and crystal structures of TpbA (Koveal et al., 2013; Xu et al., 2015), Tk-PTP (Yun et al., 2018), DUSP23a (Agarwal et al., 2008; Kuznetsov et al., 2012), and DUSP28 (Ku et al., 2017) as search models (Table 1). The molecular replacement solution obtained using the AlphaFold3-predicted model was selected for further analysis based on the log-likelihood gain (LLG) and translation-function Z-score (TFZ) values (Table 1). Model building and refinement were performed using Coot (Emsley and Cowtan, 2004) and PHENIX (Adams et al., 2010), respectively. The crystallographic statistics are presented in Table 2.

### Dephosphorylation activity assays

The wild-type construct served as a template for preparing *CCa*DUSP mutants containing D67N, C97S, or E135Q substitutions. These proteins were prepared following a procedure similar to that used for the wild-type protein. The enzymatic activity of *CCa*DUSP was measured using *para*-nitrophenylphosphate (*p*NPP) as a substrate. To determine the optimal pH for the enzymatic reaction, the initial reaction buffers containing 50 mM NaCl, 2 mM dithiothreitol, and 100 mM Bis-Tris adjusted to pH values ranging from 5.5 to 8.0 were prepared. Subsequent enzymatic analyses were performed at pH 5.5, which was optimal for this protein. For the kinetic analysis, the reaction mixture containing 20 μM recombinant wild-type or mutant *CCa*DUSP was incubated with varying concentrations of *p*NPP (0–90 mM). The reaction was carried out for 5 min and then immediately terminated by adding 80 μl of 1 N NaOH. The mixture was allowed to stand for 30 min to stabilize the chromophores. The substrate hydrolysis rate was then determined by measuring the absorbance at 405 nm using a SpectraMax Plus 384 spectrophotometer (Molecular Devices, USA) with its chamber temperature set at 30°C. To express the kinetic rates in quantitative units, the raw velocity (Abs/min) was converted to nmol/min based on the Beer–Lambert law, using the molar extinction coefficient of *p*-nitrophenol (ε = 18,000 M^-1^ cm^-1^) and the optical pathlength (b = 0.45 cm). All enzymatic assays were performed in triplicate.

## Results and Discussion

### Crystal structure of *CCa*DUSP

The crystal structure of *CCa*DUSP was determined to 2.4 Å resolution (Table 2). In the *CCa*DUSP crystal structure, a central β-sheet is composed of five β-strands [β1(Gly6–Ile9), β2(Lys12–Ser15), β3(Ala39–Ser42), β4(Met59–Leu63), and β5(Val93–His96)], which is sandwiched by six helices: four helices [α2(Thr72–Arg89), α3(Tyr101–Arg115), α4(Ala119–Ile129), and α5(Lys137–His151)] on one side and two helices [α1(Glu25–Gln35) and α^+^(Pro50–Tyr56)] on the other (Fig. 1B). The overall structure adopts the conventional DUSP fold, composed of a core region (β1–β5 and α2–α5) together with mid-domain variations (α1 and α^+^) as defined by the DUSP secondary-structure nomenclature (Fig. 1B). Structural superposition showed that *CCa*DUSP superimposes well with *P. aeruginosa* TpbA (the sole identified bacterial DUSP before this study), *T. kodakarensis* Tk-PTP (an archaeal DUSP), and human DUSP23a, with root mean square deviation (RMSD) values of 2.0 Å (over 142 aligned residues), 2.6 Å (over 145 aligned residues), and 1.2 Å (over 137 aligned residues), respectively (Fig. 1C). These results further support that *CCa*DUSP adopts a canonical DUSP fold.

### Structural analysis of the P-loop in *CCa*DUSP

DUSPs commonly possess a conserved catalytic signature, H−C−x−x−G−x−x−R−S/T, known as the phosphate-binding or P-loop motif (Tabernero et al., 2008; Tautz et al., 2013). In *CCa*DUSP, this motif resides in the β5−α3 loop and is represented by the sequence H−C^97^−R−A−G−Y−G−R^103^. Cys97 is present at the position of the catalytic nucleophile, whereas Arg103 is located at the position of the conserved arginine residue that interacts with and stabilizes the phosphate moiety of the substrate. Notably, a sulfate ion was accommodated in the active site of the *CCa*DUSP crystal structure, presumably as an analogue of the phosphate group of the substrate (Fig. 1D). This sulfate ion is stabilized by multiple hydrogen bonds with the backbone amides of Ala99, Tyr101, Gly102, and Arg103 and by electrostatic interactions with the guanidinium groups of Arg98 and Arg103 (Fig. 1E). The P-loop of *CCa*DUSP adopts a catalytically active conformation. The backbone amide groups within the loop, together with the side-chain guanidinium group of Arg103, are oriented toward the catalytic pocket, forming a positively charged surface (Fig. 1D). This electropositive environment complements the negatively charged phosphate group of the substrate and contributes to lowering the pKa of Cys97, thereby maintaining it as a reactive thiolate essential for catalysis (Kolmodin and Aqvist, 2001; Zhang, 2002; Zhang et al., 1994). This arrangement of the P-loop is highly homologous to that observed in catalytically active DUSPs, such as TpbA (PDB code 4R0S), Tk-PTP (PDB code 5Z5A), and DUSP23a (PDB code 4ERC) (Fig. 1E). Collectively, structural analyses indicated that *CCa*DUSP adopts a canonical DUSP fold with a conserved P-loop.

### Enzymatic property of *CCa*DUSP

To evaluate the phosphatase activity of *CCa*DUSP, we performed in vitro biochemical assays using *p*NPP, a widely used artificial substrate for phosphatases. Wild-type *CCa*DUSP exhibited significant dephosphorylation activity toward *p*NPP, whereas the catalytic mutant C97S showed negligible activity (Fig. 2A). Next, we examined the pH dependence of *CCa*DUSP activity and found that the enzyme exhibited optimal activity at pH 5.5 (Fig. 2B). Notably, C97S mutant displayed only background-level activity. To calculate the kinetic constants of the *CCa*DUSP dephosphorylation reactions, initial velocity data were obtained and fitted to the Michaelis–Menten equation using nonlinear regression analysis (Fig. 2C). The *k*_cat_, *K*_M_, and *k*_cat_/*K*_M_ values of *CCa*DUSP toward *p*NPP were determined to be 0.0172 min^-1^, 13.1 mM, and 1.31 M^-1^ min^-1^, respectively.

### *CCa*DUSP contains dual general acid/base residues

In addition to the conserved P-loop motif, DUSPs contain another key motif, the D-loop, which includes a conserved aspartate residue that is essential for dephosphorylation. During catalysis, this conserved aspartate residue acts as a general acid/base residue by protonating the leaving group of the substrate phosphate and subsequently activating a water molecule that hydrolyzes the phosphoenzyme intermediate (Tautz et al., 2013). Sequence (Fig. 1A) and structural alignment (Fig. 2D) showed that this aspartate residue is also conserved in *CCa*DUSP as Asp67, located in the β4−α2 loop. Interestingly, we identified another potential general acid/base residue in *CCa*DUSP, Glu135, located in the α4−α5 loop. Structural comparison suggested that this residue occupies a position analogous to the secondary general acid/base residues, Glu134 of DUSP23a (Agarwal et al., 2008; Kuznetsov et al., 2012) and Glu132 of Tk−PTP (Yun et al., 2018). To determine whether Asp67 and Glu135 serve as dual acid/base residues in *CCa*DUSP, we prepared D67N and E135Q mutants and measured their catalytic activities toward *p*NPP. Both mutations led to a substantial decrease in the dephosphorylation activity compared to that of the wild-type protein (Fig. 2E). Nonlinear regression analysis of the initial velocity data fitted to the Michaelis–Menten equation yielded the catalytic efficiency (*k*_cat_/*K*_M_) values of 0.583 M^-1^ min^-1^ and 0.422 M^-1^ min^-1^ for E135Q and D67N mutants, respectively (Fig. 2F). These values are less than half of the *k*_cat_/*K*_M_ of the wild-type protein (1.31 M^-1^ min^-1^; Fig. 2C). These results collectively demonstrate that Asp67 and Glu135 cooperate as dual general acid/base residues in the dephosphorylation reaction catalyzed by *CCa*DUSP.

## Conclusion

In this study, we determined the crystal structure of *CCa*DUSP, which adopts a canonical DUSP fold with a well-conserved catalytic cysteine residue and functions as an active phosphatase. Notably, *CCa*DUSP represents the second structurally and biochemically characterized bacterial DUSP to date and the first case of a bacterial DUSP harboring dual general acid/base residues. These findings offer insights into the catalytic diversity of bacterial DUSPs and provide a basis for future studies of DUSP family members. Another unresolved question concerns the biological functions of *CCa*DUSP. *Ca. Chx. allophototropha* is an anoxygenic phototroph that is likely exposed to fluctuations in light availability and photo-oxidative stress caused by excess light energy (Tsuji et al., 2024). Adaptation to such environmental changes may require the dynamic regulation of protein phosphorylation states, in which *CCa*DUSP may play a role. Identification of its physiological substrates and interacting partners will contribute to the elucidation of the biological function of *CCa*DUSP and the significance of its phosphatase activity in the physiology of *Ca. Chx. allophototropha*.

## Figures and Tables

**Fig. 1.**
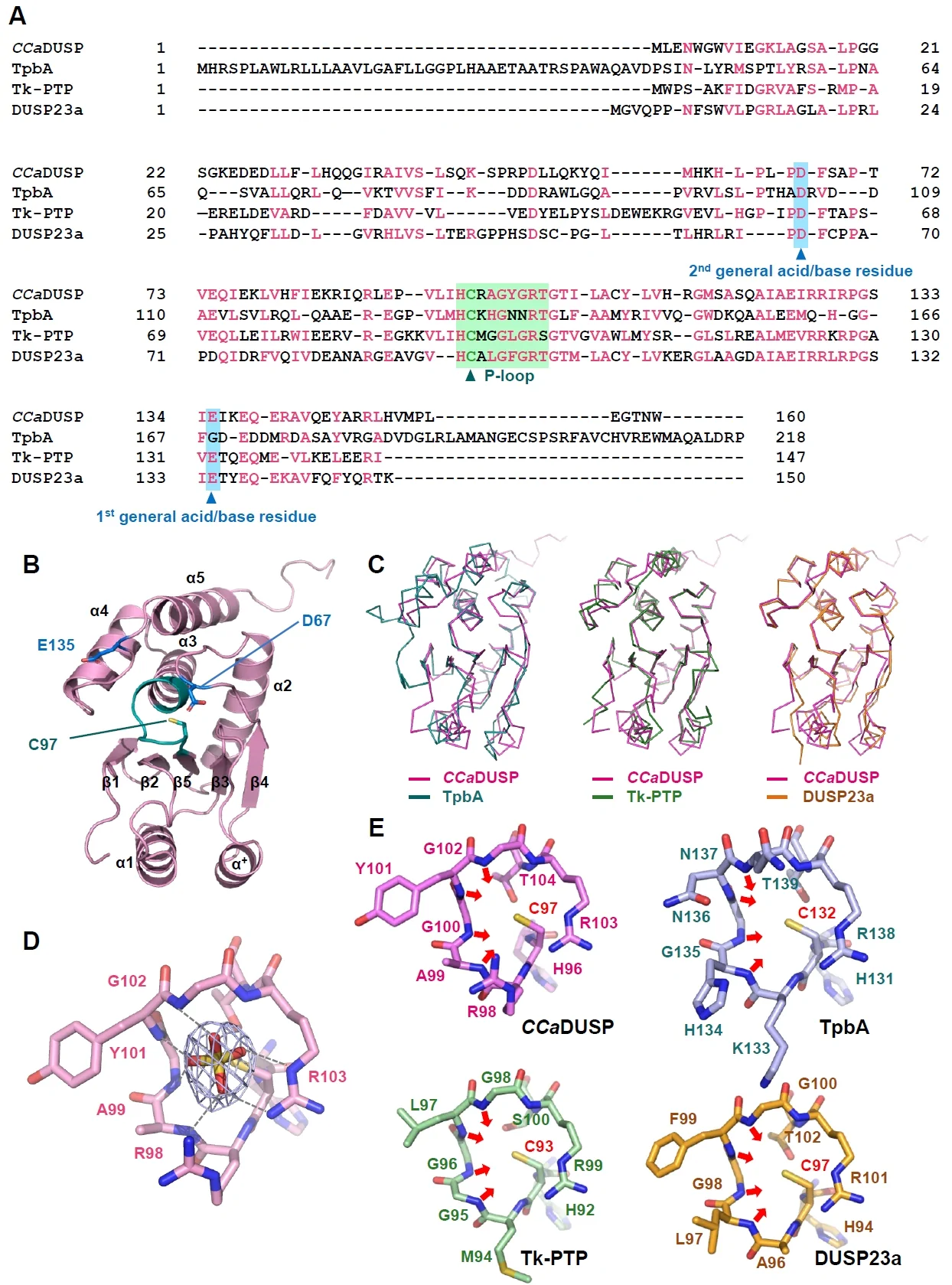
Sequence and structural analysis of *CCa*DUSP. (A) The amino acid sequences of *CCa*DUSP, TpbA, Tk-PTP, and DUSP23a were aligned using Clustal Omega. Conserved residues are highlighted in pink. The phosphate-binding loop (P-loop) and general acid/base residues are shaded in green and sky blue, respectively. The catalytic cysteine and general acid/base aspartate residues are marked with triangles. (B) Crystal structure of *CCa*DUSP. *CCa*DUSP is presented as ribbon drawings with secondary structures labeled according to their order of appearance in the primary sequence. *CCa*DUSP is colored in pink; the P-loop containing the catalytic cysteine residue (Cys97) is in green; dual general acid/base residues are in blue. (C) Structural superposition of *CCa*DUSP with TpbA (PDB code: 4R0S), Tk-PTP (PDB code: 5Z5A), and DUSP23a (PDB code: 4ERC), which are shown in C_α_ trace representation. (D) The P-loop of *CCa*DUSP is shown in sticks. A 2m*F*_o_-D*F*_c_ electron density omit map of the sulfate molecule contoured at 1.6 σ is shown together. Dashed lines indicate electrostatic interactions or hydrogen bonds mediated by the sulfate molecules. (E) The P-loops of four DUSPs are shown as sticks for comparison, as indicated. The direction of the main-chain amides of the four P-loop-constituting central residues is indicated by arrows.

**Fig. 2.**
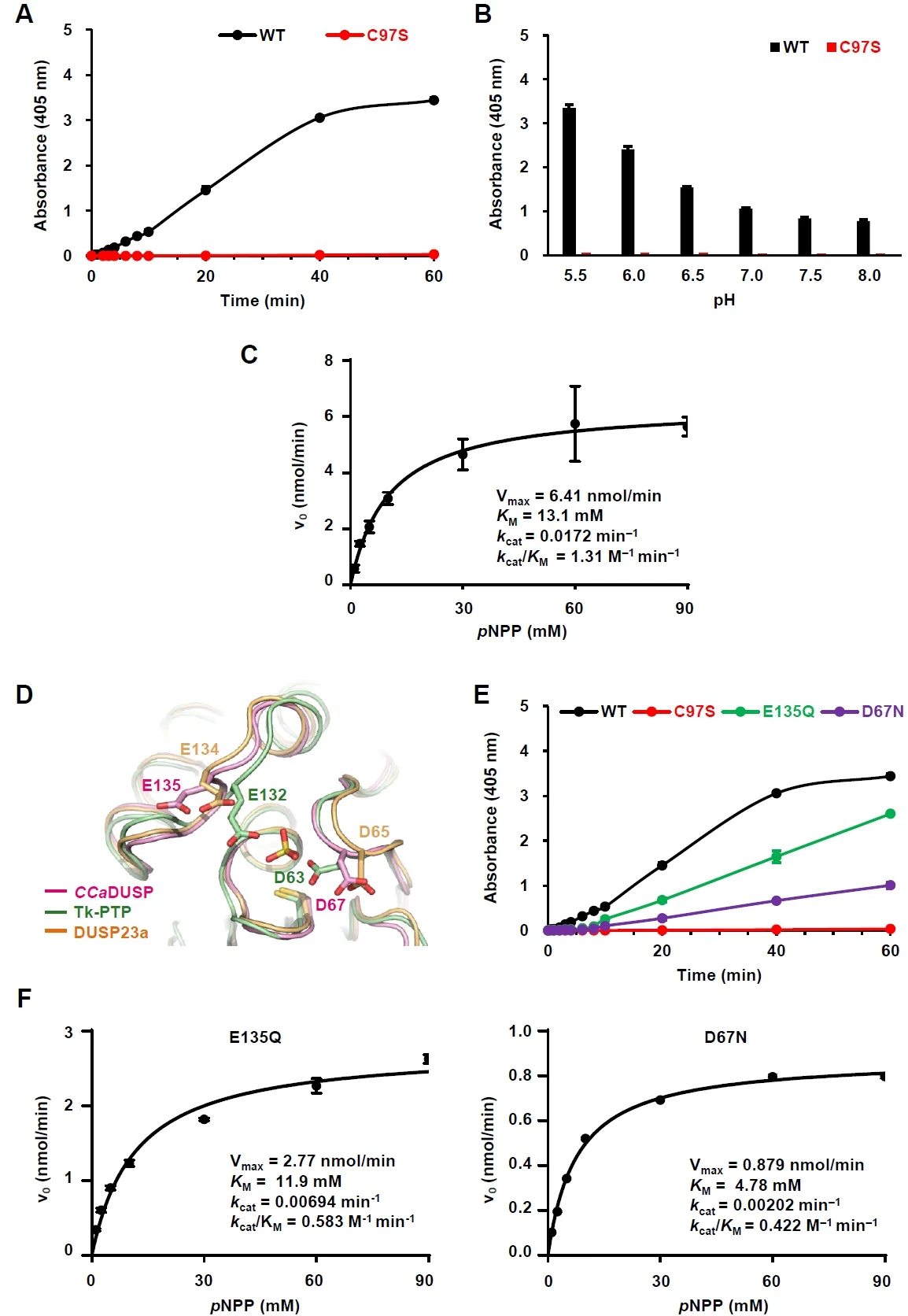
Characterization of the phosphatase activity of *CCa*DUSP. All enzymatic assays were performed in triplicate. The details of the experiments are described in the Materials and methods section. (A) Time-dependent hydrolysis of *p*NPP by wild-type and C97S mutant *CCa*DUSP was measured at 405 nm over 60 min. (B) Catalytic activity of *CCa*DUSP was measured 30 min after incubation under the indicated pH conditions using *p*NPP as a substrate. (C) Michaelis–Menten curves for wild-type *CCa*DUSP obtained by nonlinear regression analysis using GraphPad Prism. Graphs were derived from initial velocity data collected during the first 5 min of the reaction. The V_max_, *K*_M_, *k*_cat_, and *k*_cat_/*K*_M_ values are listed. (D) Structural comparison of the positions of the general acid/base residues in *CCa*DUSP, Tk-PTP, and DUSP23a. (E) Time-dependent *p*NPP hydrolysis of wild-type and D67N, C97S, or E135Q mutant *CCa*DUSP proteins. (F) Michaelis–Menten curves for D67N and E135Q mutant *CCa*DUSP proteins obtained by nonlinear regression analysis using GraphPad Prism. Graphs were derived from initial velocity data collected during the first 5 min of the reaction. The V_max_, *K*_M_, *k*_cat_, and *k*_cat_/*K*_M_ values for each protein are listed.

**Table 1.** Search models used for molecular replacement

Search model	PDB code	MR solutions	Top LLG	Top TFZ	Space group
AF3-predicted	-	1	8798.463	78.8	*P*6_3_
TpbA	4R0S	19	22.705	5.6	*P*6_4_
Tk-PTP	5Z5A	1	0.425	4.8	*P*6_1_
DUSP23a	4ERC	1	23.278	6.0	*P*6_3_
DUSP28	5Y15	30	23.634	5.6	*P*6_5_

**Table 2.** Data collection and structure refinement statistics

Data collection	PDB code 25XA
Space group	*P*6_3_
Unit cell dimensions	
a, b, c (Å)	113.50, 113.50, 66.03
a, β, γ (°)	90.00, 90.00, 120.00
Resolution (Å)	50.00−2.4 (2.44−2.40)^a^
*R*_sym_^b^	0.105 (0.452)
*R*_meas_	0.112 (0.484)
*R*_pim_	0.039 (0.170)
CC_1/2_	0.981 (0.896)
CC^*^	0.995 (0.972)
*I*/σ (*I*)	15.7 (2.9)
Completeness (%)	99.9 (100)
Unique reflections	19164 (960)
Redundancy	8.4 (8.5)
**Refinement**	
Resolution (Å)	50.00−2.40
Number of reflections	18983
*R*_work_^c^/*R*_free_ (%)	0.169/0.214
Number of atoms	
Protein	2551
Water	220
Ligand	15
Root-mean-square deviation	
Bond lengths (Å)	0.007
Bond angles (°)	0.832
Ramachandran plot (%)	
Most favored region	96.85
Additionally allowed region	3.15
Average B-values (Å^2^)	
Protein	37.0
Water	41.7
Ligand	43.1

a The numbers in parentheses are statistics from the shell with the highest resolution. ^b^*R*_sym_ = Σ |*I*_obs_ - *I*_avg_| / *I*_obs_, where *I*_obs_ is the observed intensity of individual reflection and *I*_avg_ is the average across symmetry equivalents. ^c^*R*_work_ = Σ ||*F*_o_| - |*F*_c_|| / Σ |*F*_o_|, where |*F*_o_| and |*F*_c_| are the observed and calculated structure factor amplitudes, respectively. *R*_free_ was calculated with 10% of the data.
